# Protein and chemotherapy profiling of extracellular vesicles harvested from therapeutic induced senescent triple negative breast cancer cells

**DOI:** 10.1038/oncsis.2017.82

**Published:** 2017-10-09

**Authors:** E L Kavanagh, S Lindsay, M Halasz, L C Gubbins, K Weiner-Gorzel, M H Z Guang, A McGoldrick, E Collins, M Henry, A Blanco-Fernández, P O' Gorman, P Fitzpatrick, M J Higgins, P Dowling, A McCann

**Affiliations:** 1 UCD Conway Institute of Biomolecular and Biomedical Research, School of Medicine, University College Dublin (UCD), Dublin, Ireland; 2 Systems Biology Ireland (SBI), University College Dublin (UCD), Dublin, Ireland; 3 UCD School of Medicine, College of Health and Agricultural Science, University College Dublin (UCD), Dublin, Ireland; 4 National Institute for Cellular Biotechnology, Dublin City University, Dublin, Ireland; 5 Haematology Department, Mater Misericordiae University Hospital, Dublin, Ireland; 6 UCD School of Public Health, Physiotherapy and Sports Science, University College Dublin, Dublin, Ireland; 7 Oncology Department, Mater Misericordiae University Hospital, Dublin, Ireland; 8 Biology Department, National University of Ireland Maynooth, Dublin, Ireland; 9 These authors contributed equally to this manuscript

## Abstract

Triple negative breast cancer (TNBC) is an aggressive subtype with relatively poor clinical outcomes and limited treatment options. Chemotherapy, while killing cancer cells, can result in the generation of highly chemoresistant therapeutic induced senescent (TIS) cells that potentially form stem cell niches resulting in metastases. Intriguingly, senescent cells release significantly more extracellular vesicles (EVs) than non-senescent cells. Our aim was to profile EVs harvested from TIS TNBC cells compared with control cells to identify a potential mechanism by which TIS TNBC cells maintain survival in the face of chemotherapy. TIS was induced and confirmed in Cal51 TNBC cells using the chemotherapeutic paclitaxel (PTX) (Taxol). Mass spectrometry (MS) analysis of EVs harvested from TIS compared with control Cal51 cells was performed using Ingenuity Pathway Analysis and InnateDB programs. We demonstrate that TIS Cal51 cells treated with 75 nM PTX for 7 days became senescent (senescence-associated β-galactosidase (SA-β-Gal) positive, Ki67-negative, increased p21 and p16, G2/M cell cycle arrest) and released significantly more EVs (*P*=0.0002) and exosomes (*P*=0.0007) than non-senescent control cells. Moreover, TIS cells displayed an increased expression of the multidrug resistance protein 1/p-glycoprotein. MS analysis demonstrated that EVs derived from senescent Cal51 cells contained 142 proteins with a significant increased fold change compared with control EVs. Key proteins included ATPases, annexins, tubulins, integrins, Rabs and insoluble senescence-associated secretory phenotype (SASP) factors. A fluorescent analogue of PTX (Flutax-2) allowed appreciation of the removal of chemotherapy in EVs from senescent cells. Treatment of TIS cells with the exosome biogenesis inhibitor GW4869 resulted in reduced SA-β-Gal staining (*P*=0.04). In summary, this study demonstrates that TIS cells release significantly more EVs compared with control cells, containing chemotherapy and key proteins involved in cell proliferation, ATP depletion, apoptosis and the SASP. These findings may partially explain why cancer senescent cells remain viable despite chemotherapeutic challenge.

## Introduction

In Ireland, ~2883 women will be diagnosed annually with breast cancer, representing the highest incidence of all female cancers in Ireland, with 10–15% described as triple negative breast cancer (TNBC).^1^ TNBC is characterised by its aggressive behaviour and lack of expression of the oestrogen, progesterone and HER2 receptors. This results in TNBCs not being amenable to endocrine and trastuzumab-based therapies, with conventional cytotoxic therapies such as the chemotherapeutic paclitaxel (PTX) (Taxol) the only option.^2^ TNBC is further characterised by early onset and poor prognosis,[Bibr bib3]^3^ with TNBC tumours being at a higher risk for subsequent tumour recurrence^4^ owing to its chemoresistant biology. Moreover, when TNBC relapses, it usually presents as distant metastases, which tend to have central nervous system involvement, rather than local, more treatable metastases.^5^

The chemotherapeutic PTX (Supplementary Figure 1A) preferentially binds to the beta subunit of tubulin with high affinity and enhances the polymerisation of tubulin to stable microtubules. This stabilisation means that depolymerisation cannot take place, the cells are unable to form the correct mitotic spindle and as a result cells are arrested in the G2/M phase of mitosis.^6^ Although the goal of chemotherapy is to induce tumour cell death via apoptosis, paradoxically, tumour cells can maintain viability in response to chemotherapy, by undergoing alternative fates such as therapeutic induced senescence (TIS), resulting in the ability of tumour cells to successfully evade apoptosis.^7^ Increased expression of p21, p16 and senescence-associated β-galactosidase (SA-β-Gal) activity are well-established markers of senescent cells.^8, 9^

Senescent cells although being metabolically active are also proliferatively incompetent.^10^ Therefore, chemotherapies such as PTX, with preferential efficacy on dividing cells, are less likely to induce apoptotic cell death.^11^ Moreover, senescent cells secrete the senescence-associated secretory phenotype (SASP); a secretome known to be associated with cancer-promoting phenomenon such as chronic inflammation, angiogenesis, cell proliferation and cell invasion.^12^ Importantly, Demaria *et al.*^13^ have shown using a mouse model that TIS promotes an inflammatory environment conducive to metastases and that removal of senescent cells after chemotherapy can prevent or delay cancer recurrence and metastases. Intriguingly, senescent cells produce more extracellular vesicles (EVs) than non-senescent cells facilitating their continued engagement with the *cellular milieu* both locally and distally.^14^

EVs are released by multiple cell types and can be found in blood, urine, serum and amniotic fluid.^15^ The term EVs encompasses a range of different subsets of lipid bilayer vesicles including vesicles of ~50–150 nm diameter termed exosomes. Exosomes are released by most cells upon the fusion of multivesicular bodies with the plasma membrane.^16, 17^ Exosomes are characterised by a variety of markers including the tetraspanins (CD63, CD9 and CD81), heat shock proteins (HSP70) and multivesicular body formation proteins (for example, TSG101).^18^

It is well established that tumour exosomes comprise a large population of total EVs in the blood of cancer patients.^19^ Therefore, the profiling of these EVs as circulating biomarkers in a patient’s liquid biopsy is feasible. In addition, EVs can transmit proteins, nutrients and RNA from one cell to another thereby, having a functional effect on recipient cells.^20^ Moreover, EVs have an integral role in intercellular communication in the TME,^21^ can propagate the chemoresistant phenotype and establish metastatic niches.^22, 23^ EVs also facilitate the removal of misfolded proteins or metabolic waste products that are harmful to the cell.

In relation to drug treatments and chemoresistance, EVs have been shown to neutralise targeted antibody based drugs such as trastuzumab/Herceptin, which targets HER2. Specifically, HER2-overexpressing breast carcinoma cell lines release EVs containing the HER2 protein, which preferentially sequesters trastuzumab, leading to a decreased drug concentration and attenuated interaction of trastuzumab with its intended HER2^+^ cancer cell target.^24^ EVs have also been shown to confer drug resistance in a paracrine manner through the EV-mediated transfer of the multidrug resistance protein 1/p-glycoprotein (MDR1/P-gp) from a docetaxel-resistant breast cancer cell line to its sensitive counterpart.^25^ Moreover, cancer cells can export chemotherapeutics in EVs, thereby reducing the intracellular drug concentration. In this regard, it has been shown that cisplatin-resistant ovarian carcinoma cell-derived EVs contain more cisplatin in comparison with cisplatin sensitive ovarian carcinoma cell-derived EVs.^26^

In light of the ability of chemotherapy to induce viable TIS cancer cells, and the documented preponderance of EV release from senescent compared with non-senescent cells, the overall aim of this study was to investigate the chemotherapy and protein content of EVs derived from TIS cancer cells and determine whether the resultant profiles may partially explain why cancer senescent cells remain viable despite chemotherapeutic challenge.

## Results

### PTX induces senescence in Cal51 TNBC cells

**Figure 1:**
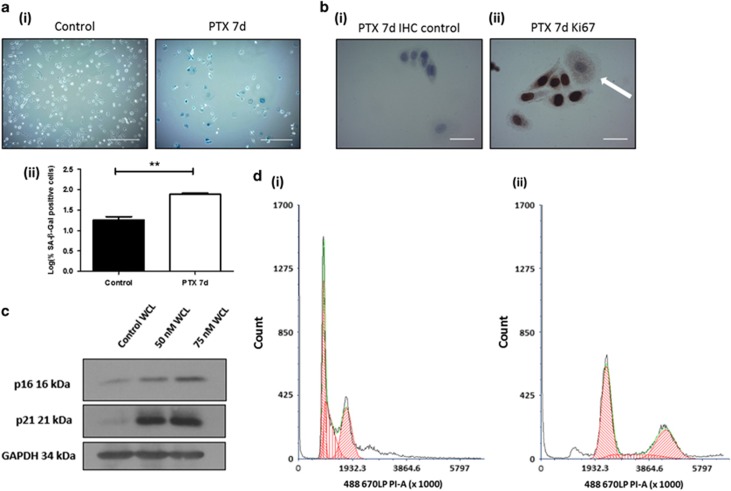
Confirmation of therapeutic induced senescence (TIS) in Cal51 triple negative breast cancer (TNBC) cells treated with 75 nM paclitaxel (PTX) for seven days. (**a**,i) Cal51 treated with 75 nM PTX for 1 week seeded at 100 000 cells/well. Cells were stained using the SA-β-Gal staining kit (Cell Signalling) with 5 mg/ml X-gal. Scale bars represent 20 μm. (**a**,ii) The percentage of positive SA-β-Gal staining was normalised to the cell count in each condition and shown in log scale (β-Gal % positivity 77%±5.204). (**b**) Cal51 TNBC cells seeded at 100 000 cells/ml, treated with 75 nM PTX for 1 week to induce senescence followed by immunohistochemical (IHC) staining for the proliferation marker Ki67, (i) negative IHC control (ii) Ki67 staining evident in proliferating cells and absent in the larger senescent cell (white arrow). Scale bars represent 50 μm. (**c**) Cal51 cells were treated with 50 nM and 75 nM PTX for 1 week. SDS–PAGE western blotting was performed using a 12% gel for p16, p21 and GAPDH. (**d**) Cal51 TNBC cells treated with PTX for 1 week undergo a G2/M cell cycle arrest. (**d**,i) Control Cal51 TNBC cell cycle profile. (**d**,ii) TIS Cal51 TNBC cell cycle profile. Cells were stained using RNase A 0.2 mg/ml and PI 50 μg/ml. All values are expressed as the mean of three independent experiments±s.e.m. The limit of ±1 skewness was set as normal distribution. Skewed data were transformed to Y=Log(Y) to fit a normal distribution. Results were analysed using the Student’s *t*-test with significant differences having a *P⩽0.05, **P⩽0.01, ***P⩽0.001.

**Figure 2:**
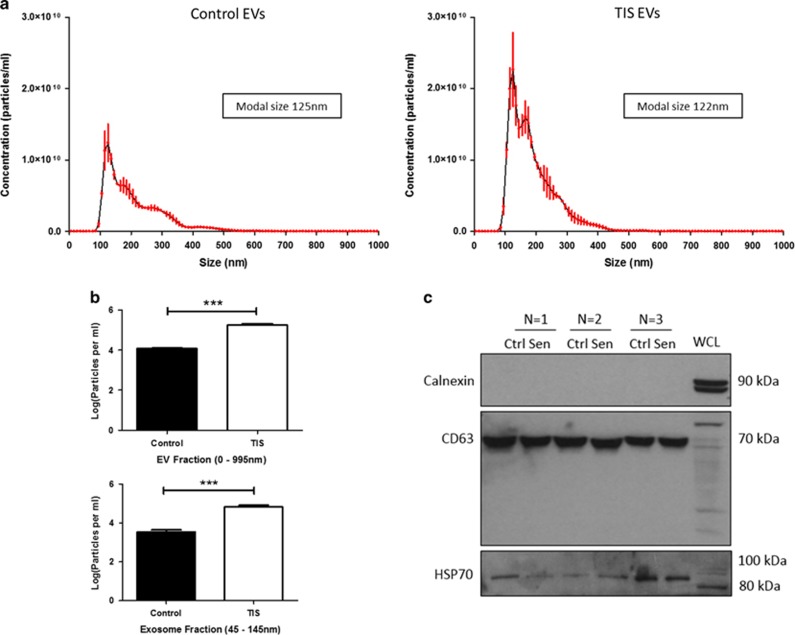
TIS Cal51 cells release significantly more EVs than control cells. (**a**) EVs were isolated using ultracentrifugation and profiles for both control and TIS EVs were recorded using NTA (NanoSight NS300 Malvern). (**b**) Particle per ml concentrations of EVs sized 0–995 nm and exosomes sized 45–145 nm were obtained using NTA and normalised to the cell count. (**c**) SDS–PAGE western blotting analyses were carried out for the EV markers CD63 and HSP70. Note, owing to the known glycosylation of CD63, band sizes of between 53 and 70 kDa are observed. Calnexin was used as a negative control as it represents an ER protein whose absence ensures for EV enrichment of endocytic origin. All values are expressed as the mean of three independent experiments±s.e.m. The limit of ±1 skewness was set as normal distribution. Skewed data were transformed to Y=Log(Y) to fit a normal distribution. Results were analysed using the Student’s *t*-test with significant differences having a *P⩽0.05, **P⩽0.01, ***P⩽0.001.

The TIS model comprised of Cal51 TNBC cells treated with 75 nM PTX for 7 days. TIS was appreciated using four routinely used markers of senescence: (1) positive SA-β-Gal activity and characteristic large flat morphology of senescent cells (Figures 1a), (2) absence of Ki67 staining (Figure 1b) (3) sodium dodecyl sulphate polyacrylamide gel electrophoresis western blot appreciation of p21 and p16 overexpression (Figures 1c) and (4) a G2/M cell cycle arrest (Figure 1d).

### TIS cells release significantly more EVs than control cells

EVs were isolated from cells by differential centrifugation unless otherwise stated. Isolation of EVs was confirmed using nanoparticle tracking analysis (NTA) measuring the concentration and size of the particles (Figure 2a). Control and TIS EVs displayed average modal sizes of 125 nm and 122 nm, respectively. TIS cells released significantly more EV (*P*=0.0002) and exosome (*P*=0.0007) fractions compared with control cells (Figure 2b). EVs were confirmed to be calnexin-negative (negative control), CD63- and HSP70-positive (positive control) by sodium dodecyl sulphate polyacrylamide gel electrophoresis western blotting (Figure 2c).

### TIS cells maintain viability via the upregulation of MDR1/P-gp

**Figure 3:**
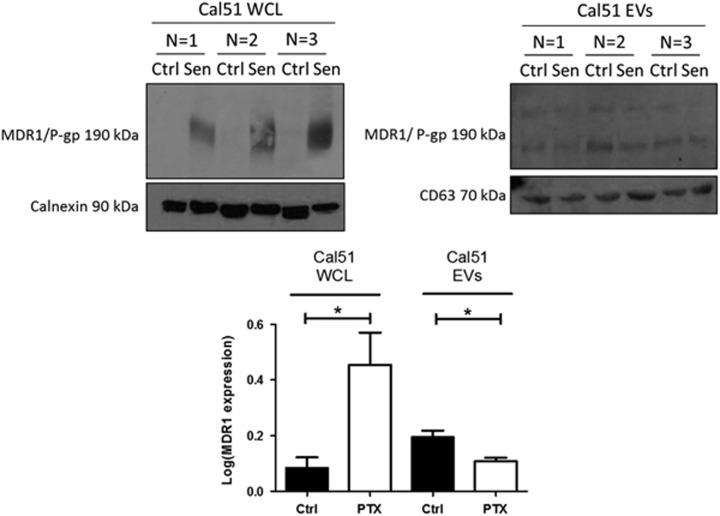
MDR1/P-gp profiling of control and therapeutic induced senescent (TIS) Cal51 cells. TIS (PTX) and non-treated control (Ctrl) Cal51 cells were SDS–PAGE western blotted for MDR1/P-gp expression. Calnexin and CD63 were used as loading controls for the whole-cell lysates (WCLs) and EVs respectively. Densitometry was undertaken using Image J software (Fiji). All values are expressed as the mean of three independent experiments±s.e.m. The limit of ±1 skewness was set as normal distribution. Skewed data were transformed to Y=Log(Y) to fit a normal distribution. Results were analysed using the Student’s *t*-test with significant differences having a *P⩽0.05, **P⩽0.01, ***P⩽0.001.

The TIS model was further investigated for the expression of the multidrug resistance/p-glycoprotein (MDR1/P-gp). TIS Cal51 cells treated with 75 nM PTX for 7 days resulted in a significant increased expression of MDR1/P-gp in the whole-cell lysates, compared with control Cal51 cells (*P*=0.04). For the EV fraction, those derived from TIS cells demonstrated lower amounts of MDR1/P-gp compared with those derived from control Cal51 cells (*P*=0.04) (Figure 3).

### TIS cells maintain viability via the removal of key proteins in their EVs

**Figure 4:**
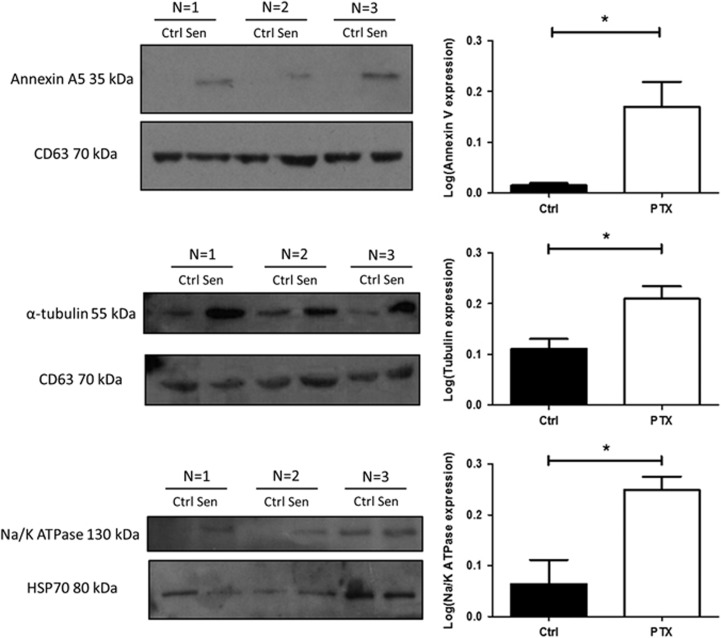
SDS–PAGE western blot validation of the increased abundance of Annexin V, Na/K ATPase and α-tubulin in the EVs derived from TIS Cal51 cells compared with EVs derived from non-senescent non-treated controls. EVs isolated from TIS and control cells were immunoblotted to validate the protein expression of annexin V, Na/K ATPase and α-tubulin from the MS data. CD63 and HSP70 were used as loading controls. Densitometry was completed using Image J software (Fiji). All values are expressed as the mean of three independent experiments±s.e.m. The limit of ±1 skewness was set as normal distribution. Skewed data were transformed to Y=Log(Y) to fit a normal distribution. Results were analysed using the Student’s *t*-test with significant differences having a *P⩽0.05, **P⩽0.01, ***P⩽0.001.

A proteomic study of the contents of EVs isolated from TIS and control cells, demonstrated that EVs derived from TIS Cal51 cells contained 142 proteins with a significant increased fold change compared with EVs derived from non-senescent cells (Supplementary Table 1). Key proteins of interest shown to be significantly higher in abundance in EVs from TIS Cal51 cells compared with control cells are displayed in Table 1. These were annexin V; which has a role in apoptosis, chemoresistance and metastasis, Na/K ATPase; an ion channel involved in the depletion of cellular ATP levels and α-tubulin; a central part of the cytoskeleton targeted by PTX. Western blot analyses confirmed a significant increased abundance of Annexin V (*P*=0.03), Na/K ATPase (*P*=0.03) and α-tubulin (*P*=0.04) in EVs derived from senescent Cal51 cells compared with EVs derived from control cells, corroborating the mass spectrometry (MS) data (Figure 4). The EV proteomic analysis also identified higher levels of insoluble SASP proteins in the EVs derived from TIS cells compared with controls (Table 1). Specifically, fibronectin, collagens alpha-1 (V) and alpha-2 (IV) and laminin subunits alpha-5 and beta-1.^27^

**Figure 5:**
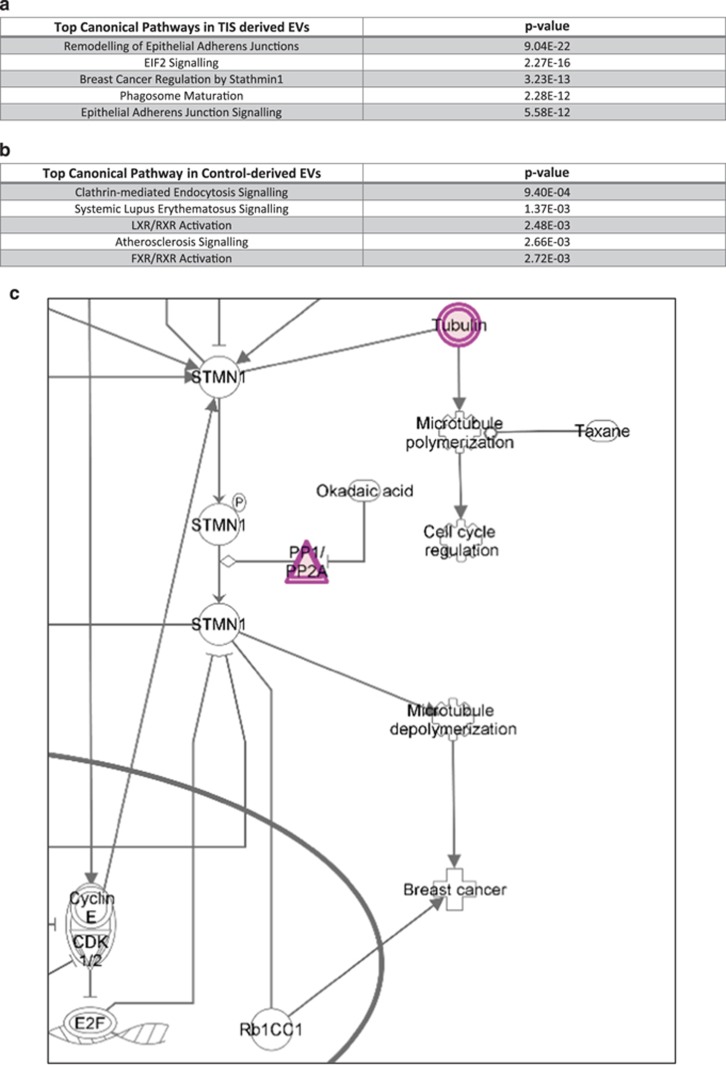

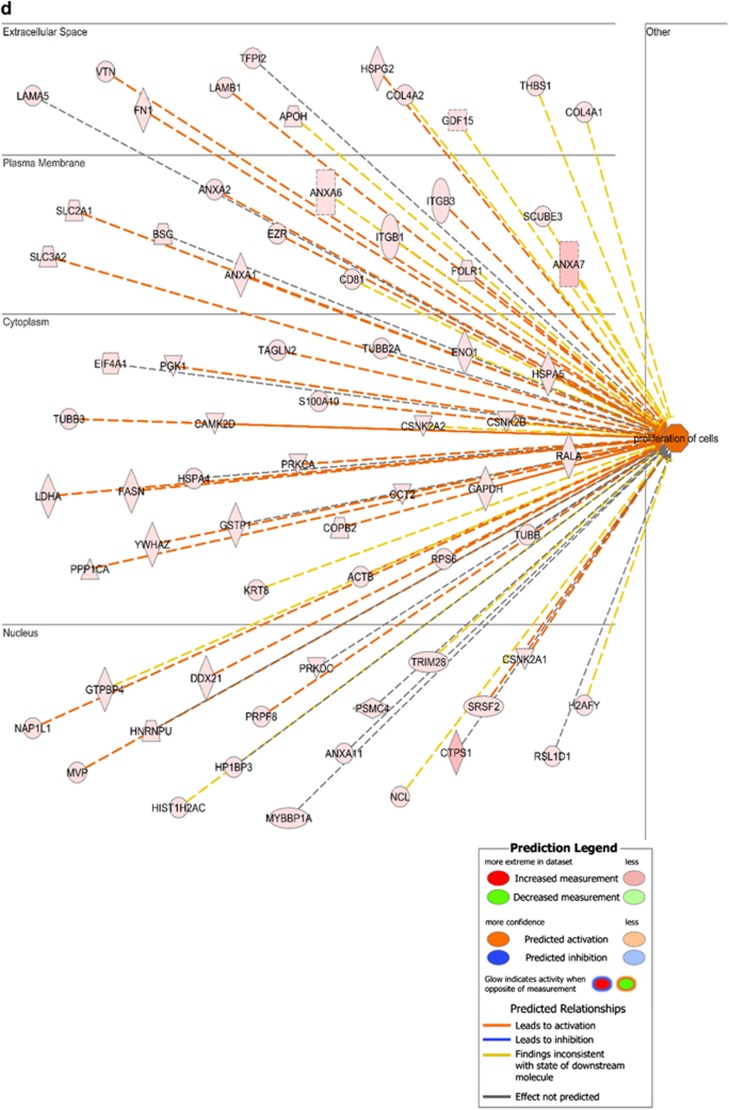
Pathway analysis of proteins in higher abundance in TIS EVs. (**a**) The top overrepresented pathways in TIS EVs by IPA. (**b**) The top overrepresented pathways in control EVs by IPA. (**c**) Proteins from the data set (red) present in TIS EVs overlaid on the ‘*Breast Cancer Regulation by Stathmin 1*’ pathway created by IPA. (**d**) Sixty-nine proteins from the data set (red) present in TIS EVs associated with cell proliferation, generated by IPA. *P*-values reported for IPA results are calculated by IPA using a right-sided Fisher exact test for over-representation analysis and Benjamini–Hochberg correction for multiple hypothesis testing correction.

Pathway analysis of the contents of the TIS EVs using Ingenuity pathway analysis (IPA) revealed the following pathways; remodelling of epithelial adherans junctions, EIF2 signalling, breast cancer regulation by stathmin 1 (Figures 5a and c, Supplementary Figure 2), phagosome maturation and epithelial adherens junction signalling pathways. Interestingly, InnateDB database analysis demonstrated that the stathmin and breast cancer resistance to anti-microtubule agent’s pathway was the second most common pathway in which the proteins from our data set are involved. Pathway analysis of the contents of control EVs using IPA identified the following pathways; clatherin-mediated endocytosis signalling, systemic lupus erythematosus signalling, LXR/RXR (liver X receptor/retinoid X receptor) activation, atherosclerosis signalling and FXR/RXR (farnesoid X receptor/retinoid X receptor) activation (Figure 5b). IPA of proteins present in a higher abundance in EVs derived from TIS Cal51 cells revealed that 69 out of 142 proteins are involved in cell growth and proliferation. The 69 proteins displayed a common function as predicted activators of cell proliferation (Figure 5d).

### TNBC cells treated with the PTX analogue Flutax-2, release EVs containing Flutax-2

**Figure 6:**
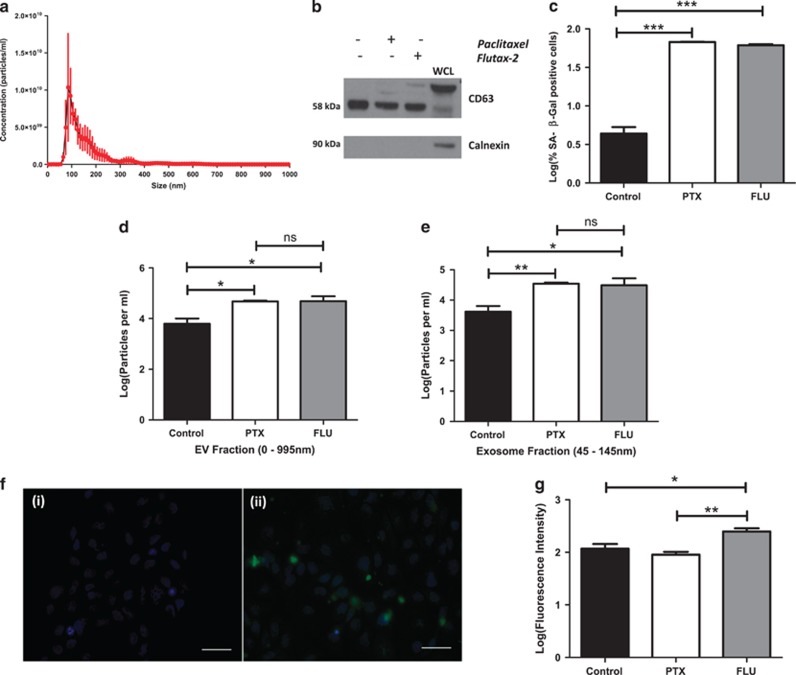
Fluorescence Intensity levels of the paclitaxel analogue Flutax-2 in EVs derived from TIS Cal51 cells. (**a**) Nanoparticle tracking analysis (NTA) confirming EV isolation using Exospin. (**b**) Western blot confirmation of positive CD63 and negative calnexin markers. Note, owing to the known glycosylation of CD63, band sizes of between 53 and 70 kDa are observed. (**c**) Quantification of positive SA-β-Gal staining in control versus PTX treated and Flutax-2 (FLU)-treated cells. (**d**) Particle per ml concentration of EVs sized 0–995 nm was obtained using NTA and normalised to the cell count. (**e**) Particle per ml concentration of exosomes sized 45–145 nm was obtained using NTA and normalised to the cell count. (**f**) Fluorescent images of (i) control versus (ii) Flutax-2 (FLU)-treated cells showing Flutax-2 present within the cytoplasm. Images were taken with a × 40 oil objective on a Leica DMI6000B epifluorescent microscope. Individual colour channels were merged and pseudocolours were applied with Image J (Fiji) software. Any contrast adjustments were applied strictly across all channels in both images. Scale bars represent 50 μm. (**g**) Graphical representation of the differing fluorescence levels detected in EVs derived from control, PTX treated and Flutax-2 (FLU) treated Cal51 TNBC cells. In relation to the concentration of Flutax-2, the manufacturers recommend using × 10 the concentration of paclitaxel (75 nM), therefore, 750 nM was used. https://www.thermofisher.com/order/catalog/product/P22310. All values are expressed as the mean of three independent experiments±s.e.m. The limit of ±1 skewness was set as normal distribution. Skewed data were transformed to Y=Log(Y) to fit a normal distribution. Results were analysed using the Student’s *t*-test, with significant differences having a *P⩽0.05, **P⩽0.01, ***P⩽0.001.

Flutax-2 contains an Oregon Green 488 fluorescent label attached to the 7’-carbon of the PTX, which permits selective binding of the probe to microtubules (Supplementary Figure 1B). Cells were treated with either 75 nM PTX or 750 nM Flutax-2. Nanoparticle tracking analysis (NTA) (Figure 6a) and western blot analyses confirmed enrichment for EVs (Figure 6b), SA-β-gal quantification confirmed successful senescence induction (Figure 6c). NTA quantification demonstrated a significant increase in the particles per ml of both EVs (*P*=0.03) (Figure 6d) and exosome subfractions (*P*=0.04) (Figure 6e) isolated from Flutax-2 and PTX-treated cells compared with non-treated control cells. EVs were subsequently sonicated to break open the membranes allowing fluorescence to be read on a plate reader at 488 nm. Fluorescent microscopy confirmed Flutax-2 uptake into Cal51 cells (Figure 6f). Figure 6g demonstrates significantly higher levels of fluorescence detected in EVs derived from Flutax-2-treated cells.

### Treatment of Cal51 TNBC cells with the exosome biogenesis inhibitor GW4869

**Figure 7:**
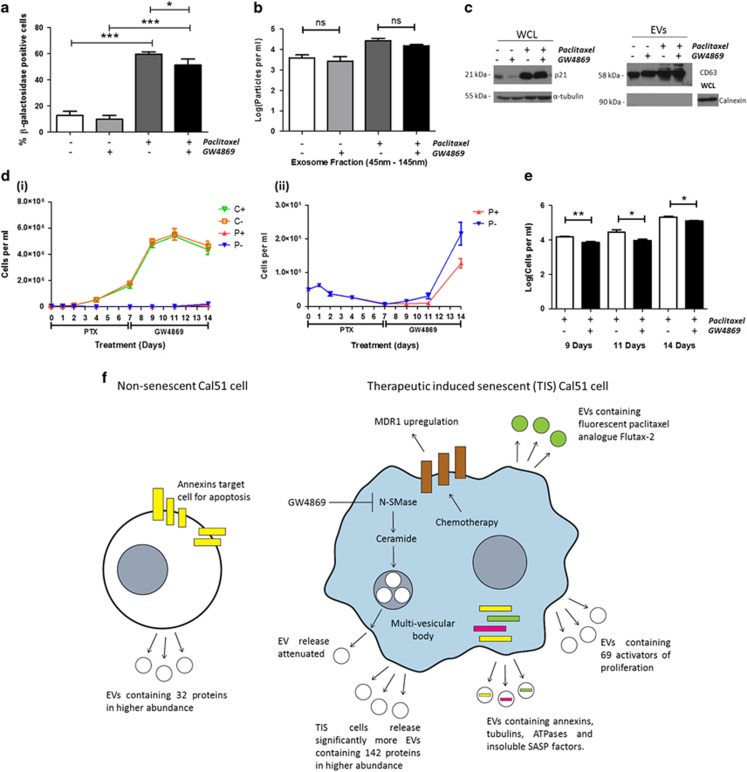
Treatment of TIS Cal51 cells with the exosome biogenesis inhibitor GW4869 results in a reduction in the number of SA-β-Gal-positive cells and reduced growth potential. (**a**) Following 7 days culture of the Cal51 cells in control or senescent inducing conditions (PTX 75 nM), the cells were then treated with control vehicle or 75 nM PTX and control vehicle or 5 μm GW4869 for 48 h. Cells were seeded at 100 000 cells/well and stained using the SA-β-Gal staining kit (Cell Signalling) with 5 mg/ml X-gal. The percentage of positive SA-β-Gal staining was normalised to the cell count in each condition. (**b**) Nanoparticle tracking analysis was carried out on each exosome sample isolated using Exospin and normalised to the cell count. (**c**) Western blot appreciation of p21 expression on whole-cell lysates (WCLs) and EV confirmation using CD63-positive and calnexin-negative expression. (**d**,i) Growth curve of all four conditions (control plus GW4869 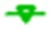
C+, control minus GW4869 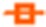
C−, PTX plus GW4869 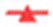
P+ and PTX minus GW4869 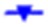
P−) over a 14-day time course. (ii) Expanded representation of (**d**,i) PTX plus GW4869 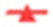
P+ and PTX minus GW4869 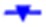
P− over a 14-day time course. (**e**) Bar chart comparing cell counts of PTX minus GW4869 and PTX plus GW4869 treatments on days 9, 11 and 14. (**f**) **Proposed Model:** Cancer cells treated with chemotherapy signal apoptosis on their cellular membrane via the annexin family of proteins and die. In contrast, therapeutic induced senescent (TIS) cells maintain viability via an upregulation of MDR1/P-gp causing the efflux of chemotherapy, the release of substantially more EVs than control cells containing annexins, tubulins, ATPases, insoluble SASP factors, 69 activators of proliferation and discarded chemotherapy (Flutax-2). All values are expressed as the mean of three independent experiments±s.e.m. The limit of ±1 skewness was set as normal distribution. Skewed data were transformed to Y=Log(Y) to fit a normal distribution. Results were analysed using the Student’s *t*-test, with significant differences having a *P⩽0.05, **P⩽0.01, ***P⩽0.001.

Cal51 cells were treated ±75 nM PTX for 7 days. Cells were then treated for a further 48 h ±GW4869. Treatment of TIS cells with GW4869 lead to a significant reduction in SA-β-gal staining (*P*=0.04) compared with TIS cells treated with control vehicle alone (Figure 7a). There was a non-significant trend (*P*=0.12) towards a reduction in the number of exosomes released from TIS cells treated with GW4869, gating specifically the 45–145 nm size (Figure 7b). TIS cells maintained p21 upregulation in response to GW4869 and EV isolation was confirmed using western blot analyses (Figure 7c).

Figure 7d (i) represents a growth curve of all four conditions (control plus GW4869, control minus GW4869, PTX plus GW4869 and PTX minus GW4869) over a 14-day time course. PTX treatment for 7 days induced TIS in Cal51 cells. These cells were subsequentually treated with GW4869 on day 7 and cells were enumerated on days 9, 11 and 14 following this treatment. GW4869-treated TIS cells displayed a significant reduction in cells per ml on days 9 (*P*=0.007), 11 (*P*=0.04) and 14 (*P*=0.05) compared with TIS cells treated with control vehicle alone (Figure 7d (ii) and E).

In summary, PTX induces senescence in Cal51 TNBC cells with a concomitant upregulation of MDR1/P-gp. TIS cells release significantly more EVs compared with control cells, which not only contain the PTX analogue (Flutax-2) but in addition, key proteins involved in cell proliferation, ATP depletion, apoptosis and the SASP. The exosome biogenesis inhibitor GW4869 results in a reduction in the number of SA-β-gal-positive cells and lowers the proliferation rate of TIS cells (Figure 7f).

## Discussion

Chemotherapy such as doxorubicin, has been shown to induce TIS in both p53 wild-type and p53 mutant cell lines.^28^ Here we demonstrate induction of TIS in the p53 wild-type TNBC cell line Cal51 using the chemotherapeutic PTX. Clinically, TIS occurs in breast tumour biopsies from patients who received neoadjuvant chemotherapy were 41% positive for SA-β-Gal activity.^29^ This suggests that TIS is a novel detrimental mechanism of breast cancer cell survival against chemotherapy. Our data show that the MDR1/P-gp drug efflux pump is upregulated in TIS cells accompanied with a marked increase in p21 expression. Similar to previous studies MDR1/P-gp is detectable in our EV fractions with lower levels in TIS EVs, possibly referring to the retention of this efflux pump by the parent cells in their chemotherapeutic stressed condition. Mechanistically, it is known that the E2F-associated phosphoprotein stimulates the MDR1/P-gp promoter, leading to the upregulation of MDR1/P-gp in a p21-dependent manner.^30^ Interestingly, MDR1/P-gp has also been shown to transfer resistance to docetaxel via EVs in breast and prostate cancer cells.^25, 31^

The higher abundance of EVs in the blood of breast cancer patients with stage IV disease has previously been reported.^32^ Our study has found that TIS cells release significantly more EVs than control cells, suggesting that increased EV release is a mechanism conducive to cell survival, following chemotherapeutic cell stress. This is corroborated by Lehmann *et al.*^14^ who showed that irradiation-induced senescent prostate cancer cells released more EVs than controls.^4^

We hypothesise that the novel ability of cancer senescent cells to remove ATPases, tubulins, Rabs, insoluble SASP factors, integrins and annexins via EVs, in part conserves the senescent phenotype. Intriguingly, 69 of 142 proteins in higher abundance in TIS EVs are predicted activators of cell growth and proliferation, suggesting that the removal of these proteins by TIS cells represents a further potential mechanism to maintain their senescent phenotype.

Our data show eight ATPases present in higher abundance in TIS EVs compared with controls, including the 26S protease regulatory subunits 6B and 10B (Table 1). The significance of these subunits’ abundant expression in TIS EVs is potentially important, implying a mechanism for retaining maximum ATP levels for the survival and metabolic needs of the senescent cell. Moreover, a sodium/potassium ATPase subunit displayed the highest abundance in the MS profiles for the TIS EVs. This ATPase enables the hydrolysis of ATP,^33^ suggesting that its removal via EVs may potentially conserve vital intracellular ATP resources crucial for maintaining the metabolic demands of senescent cells and therefore integral for senescent cell survival.^34^

In addition, our results show an increased abundance of eight annexins within TIS EVs compared with controls (Table 1). We hypothesise that annexin removal in EVs increases the survival of TIS cells via their interactions in apoptotic, chemoresistant and metastatic pathways. Specifically, Annexins A1 and A5 are inhibitors of phospholipase A2 signalling,^35, 36^ Annexin A5 is a marker of apoptosis,^37^ whereas Annexin A6, A11 and A2 are involved with cancer aggressiveness promoted by EVs,^38^ chemoresistance^39^ and EV-mediated angiogenesis and metastasis,^40^ respectively. In summary, therefore, removal of annexins via EVs is potentially advantageous to TIS survival.

Our data (Table 1) demonstrated an increase in nine different subunits of tubulin within TIS EVs compared with controls. Pathway analyses showed that the 'stathmin and breast cancer resistance to anti-microtubule agents’ pathway' is upregulated in TIS EVs. Normally, during mitosis, microtubule polymerisation occurs, which is crucial for cell cycle progression. However, stathmin promotes depolymerisation of microtubules. Differential expression levels of stathmin leads to G2/M arrest.^41^ Interestingly, protein phosphatase 2A and PP1 (protein phosphatase 1) regulate the phosphorylation of stathmin,^42^ with PP1 present in higher abundance in TIS EVs compared with controls (Supplementary Table 1). Of note, the regulatory subunit 15B of protein phosphatase 1 has been identified as a survival factor in breast cancer.^43^ A recent study demonstrated that non-small cell lung cancer cells, resistant to PTX, have high expression of stathmin and inhibition of stathmin expression increased sensitivity to this chemotherapeutic.^44^ Potentially therefore, the removal of tubulin and PP1 (stathmin regulator) from TIS cells, could suggest that microtubule dynamics have been switched from the stabilisation effect of PTX to the depolymerisation effect of stathmin, crucial in maintaining G2/M arrest and the chemoresistant and senescent phenotypes.

In addition, there was an increase in three integrin subunits; integrin beta-3, integrin beta-1 and integrin alpha-V, within TIS EVs compared with controls. Interestingly, integrin beta-3 has been associated with the progression of glioblastoma^45^ with attenuated integrin beta-3 expression resulting in glioblastoma cells becoming senescent.^46^ The integrin alpha-V-beta-3 complex regulates cancer cell survival and proliferation by signalling through the ERK1/ERK2 MAPK pathway in MDA-MB-231 breast cancer cells.^47^ Moreover, a recent study has shown that integrin beta-3 is a marker and regulator of senescence, activating the TGF-β pathway leading to senescence in human primary fibroblasts. Downregulation of integrin beta-3 has also been shown to result in the reduction in oncogene-induced senescence and TIS.^48^ Overall therefore, TIS cells may potentially remove the integrin alpha-V-beta-3 complex in EVs to maintain the senescent phenotype.

There was also an increase in 6 Rab proteins within TIS EVs compared with controls (Table 1). Rabs modulate the release of EVs from the multivesicular bodies. Rab-mediated EV trafficking is important for migration, invasion and proliferation.^49^ K562 leukaemic cells express higher levels of Rab11 compared with other Rabs, previously shown to modulate EV release.^50^ The increased release of EVs containing these Rabs suggests a possible link to the increased release of EVs compared with control cells.

In relation to the SASP, it is known that the insoluble components can affect the physical properties of tissue structure relaxing the extracellular matrix support. Moreover, the soluble factors of the SASP *in vivo,* potentially allow tumour cells to migrate through the extracellular matrix and metastasise.^51^ Of note, the insoluble SASP factors (fibronectin, collagen alpha-1(IV) chain, collagen alpha-2(IV) chain, laminin subunit alpha-5 and laminin subunit beta-1) (Table 1)^53^ were identified in EVs from TIS Cal51 cells. Therefore, our findings support the proposal that EVs derived from TIS cells not only represent a mechanism of trafficking out unwanted cellular cargo but could equally be utilising EVs to transport senescence/tumour promoting proteins to neighbouring/distant sites to further promote the senescent/tumorigenic phenotype. Urbanelli *et al.*^52^ similarly suggest a role for EVs in the maintenance and promotion of the senescent phenotype via the SASP.

MS analysis of EVs from control cells identified 32 proteins with significantly increased fold change when compared with TIS EVs (Supplementary Table 2). We hypothesise that these proteins are crucial for TIS cell survival and therefore not released in EVs. Two key pathways in higher abundance in control EVs were LXR/RXR and FXR/RXR activation. Potentially, activation of these pathways is crucial for the survival of TIS cells. LXR activation is known to inhibit proliferation of breast cancer cells^53^ and FXR activation in breast cancer cells results in cytotoxicity.^54^ Activated FXR can also counteract the tumour promoting ability of cancer-associated fibroblasts within the breast cancer microenvironment.^55^ Therefore, one might hypothesise that the removal of LXR and FXR pathway components in control cell EVs suggests that normal proliferation occurs. In contrast, TIS cells retaining these components may allow them to maintain replicative stasis, maintain senescence and evade apoptosis by chemotherapy.

Cal51 cells treated with the fluorescent PTX analogue Flutax-2 demonstrated removal of chemotherapy in EVs. The significantly higher detected fluorescence in EVs derived from Flutax-2-treated cells indicates that the chemotherapy is present within the EVs, demonstrating a potential novel method by which senescent cancer cells can remove chemotherapy. Whether this packaging and removal of chemotherapy by the cells is a passive or active process remains to be elucidated.

Previously, it has been shown that treatment with GW4869 impairs EV formation and subsequent release from macrophages.^56^ In our study, TIS cells treated with GW4869 showed significant reduction in SA-β-gal activity a key marker of senescence. We also speculate that TIS cells utilise exosome biogenesis to maintain their proliferative potential, as blocking this with GW4869 lead to a reduction in proliferative recovery. The lack of change in p21 following GW4869 treatment for 48 h may reflect a time-dependent observation.

In conclusion, our study demonstrates that TIS cells release significantly more EVs than control cells. Importantly, these EVs not only contain Flutax-2 (PTX analogue) but also suggests a novel mechanism by which TIS cells remove key proteins involved in cell proliferation, ATP depletion, apoptosis and the SASP. These findings may partially explain why cancer senescent cells remain viable despite chemotherapeutic challenge and indeed can continue to interact with the tumour microenvironment both locally and distally.

## Materials and methods

### Cell culture

Cal51 TNBC cell line (p53 wild type) (DSMZ, Braunschweig, Germany, #ACC 302) was cultured in Dulbecco's Modified Eagle's Medium (Lonza, Visp, Switzerland, #BE12-614 F) supplemented with 10% (v/v) FBS (foetal bovine serum) (Gibco, Thermo Fisher Scientific, Waltham, MA, USA, #10270-106) and 1% (v/v) sodium pyruvate (Gibco, Thermo Fisher Scientific, #S8636-100 ML) at 37 °C in a humidified atmosphere of 5% CO_2_. Cal51 cells were authenticated by DDC Medical, Fairfield, OH, USA in 2015 and routinely tested negative for mycoplasma contamination.

### PTX induced senescence

Cells were treated for 7 days with 75 nM PTX (Sigma-Aldrich, St Louis, MO, USA, #T7402-1MG). Senescence was confirmed through appreciation of SA-β-Gal activity, Ki67-negative immunohistochemistry staining, p21 and p16 protein expression using western blot analysis and cell cycle evaluation using flow cytometry.

### Flutax-2

‘Flutax-2’, an Oregon Green 488 PTX conjugate (Supplementary Figure 1B) (Thermo Fisher Scientific, #P22310), fluorescently labels the tubulin cytoskeleton. Cal51 cells were treated for 5 days with 75 nM PTX to induce senescence. On day 5, the treatment was changed to EV-depleted-serum medium with 750 nM Flutax-2 for 48 h. This 10 × higher concentration of Flutax-2 compared with PTX (75 nM) is the recommended dose as per manufacturers recommendations. EVs were isolated using Exospin, (Cell Guidance Systems, Cambridge, UK, #EX-01) characterised and profiled using NTA (NanoSight NS300; Malvern, Malvern, UK) with EV Flutax-2 fluorescence quantified at 488 nm on a microplate reader (Molecular Devices, Spectramax, Sunnyvale, CA, USA).

### SA-β-Gal staining assay

TIS cells were seeded at 1 × 10^5^ cells/ml, cultured for 24 h, fixed and stained using the SA-β-Gal staining kit, 5 mg/ml (Cell Signalling Technology, Danvers, MA, USA, #9860), incubated for 16 h, imaged using a light microscope (Olympus model CKX41, Tokyo, Japan) and enumerated for SA-β-Gal positivity using Image J software (FIJI).

### Immunohistochemistry for cultured cell lines

Cells were seeded at 1 × 10^5^ cells/ml, fixed with ice-cold methanol (Sigma-Aldrich, #24229-2.5L-R) and stained using the Novolink Polymer Detection (Leica Biosystems, Wetzlar, Germany, #RE7140-CE). Cells were stained for Ki67 (1:1000) (Thermo Fisher Scientific, #MA5-15690), counterstained using haematoxylin (Reagecon, Shannon, County Clare, Ireland, #RBA-4201-00A), mounted onto slides using Mowiol 4–88 (Sigma-Aldrich, #81381) and imaged with a × 60 oil immersion lens (Nikon Eclipse E600 microscope, Tokyo, Japan).

### Cell cycle assay

A total of 1 × 10^6^ cells/ml were fixed in 1 ml of ice-cold 70% methanol (Sigma-Aldrich, #24229-2.5L-R), washed in PBS (phosphate-buffered saline) (Oxoid, Basingstoke, Hampshire, UK, #BR0014G), treated with RNAse 0.2 mg/ml (Sigma-Aldrich, #R6513-10MG) and stained with propidium iodide 50 μg/ml (Molecular Probes Life Technologies, Eugene, OR, USA, #P1304MP) for 30 min at 37 °C with agitation. The cells were washed once more with PBS and resuspended in 500 μl PBS. Samples were analysed using a BD Accuri C6 (BD Biosciences, San Jose, CA, USA) with the default configuration, 20 000 single cells were recorded per sample and the results were analysed using DeNovo Software FCS Express RUO Version 6.

### SDS–PAGE and western blotting

Sodium dodecyl sulphate polyacrylamide gel electrophoresis and western blotting were performed as described previously.^57, 58^ The following antibodies were used: p16 (Santa Cruz Biotechnology, Dallas, TX, USA, #sc-56330), MDR1/P-gp (Santa Cruz Biotechnology, #sc-1517), calnexin (Santa Cruz Biotechnology, #sc-80645), α-tubulin (Santa Cruz Biotechnology, #sc-32293), annexin V (Abcam, #ab14196), CD63 and HSP70 (System Biosciences, Palo Alto, CA, USA, #EXOAB-CD63A-1), p21 (BD Biosciences, #556430), GAPDH (Cell Signalling, #5174P) and Na/K ATPase (Novus Biologicals, Littleton, CO, USA #NB300-146). The differential CD63 bands potentially represents either differential glycosylation^59^ or the isolation method used to harvest EVs.^60^ Densitometry was completed to determine the expression of proteins. Densitometry was completed using Image J software with all results normalised to the loading control.

### EV isolation

Cells grown for EV harvesting purposes were cultured prior to isolation in media supplemented with bovine EV-depleted FBS for 48 h. FBS was depleted of EVs by ultracentrifugation at 120 000 × *g* for 16 h at 4 °C. The RCF average was calculated using the Beckmann Coulter rotor calculator for the Beckman Coulter Optima L-100 XP Ultracentrifuge and Beckman Coulter Type 70Ti rotor. EV-depleted FBS media was conditioned for 48 h then collected and centrifuged at 300 × *g* at 4 °C for 10 min followed by 2000 × *g* at 4 °C for 20 min to ensure cellular debris removal. The supernatant was filtered using a 0.22 μm syringe filter (Millipore, Billerica, MA, USA, #SLGP033RS). For isolation using the ultracentrifugation protocol, the supernatant was sealed in an ultracentrifugation tube (Beckman Coulter, Brea, CA, USA, #342414), and subsequently ultracentrifuged twice at 120 000 × *g* for 1 h 15 min at 4 °C. For isolation using Exospin the manufacturer’s guidelines were followed (Cell Guidance Systems, #EX-01). EV counts represent values normalised to cell count.

### NanoSight NS300 EV profiling

EV samples were run using the standard measurement procedure at 25 °C with a constant syringe infusion rate of 50 as per the NanoSight NTA 3.1 Software (Malvern). The data for each sample were obtained from 10 independent 60 s video captures on the NS300, analysed and normalised to final cell counts for each condition.

### Quantitative proteomic profiling by label-free liquid chromatography–MS/MS analysis

EV pellets were solubilised in 8 m urea/50 mM NH4HCO3/0.1% ProteaseMax using sonication, with resultant protein levels quantified using a Bradford assay. Following dithiothreitol reduction and iodoacetic acid-mediated alkylation, a double digestion was performed using Lys-C (for 4 h at 37 °C) and Trypsin (overnight at 37 °C) on 5 μg of protein. The samples were desalted prior to analysis using C18 spin columns (Thermo Scientific) and 500ng was loaded onto an Ultimate 3000 NanoLC system (Dionex Corporation, Sunnyvale, CA, USA) coupled to a Q-Exactive mass spectrometer (Thermo Fisher Scientific). The raw data were analysed using Progenesis QI for Proteomics software (version 3.1; Non-Linear Dynamics, a Waters company, Newcastle upon Tyne, UK) as previously described,^61^ with following modifications: the filter mass peaks with charge states from+1 to+6 was applied to the MS/MS data files, peptides were identified using taxonomy: Homo sapiens in the SwissProt database and peptides with XCorr scores>1.9 for +1 ions,>2.2 for +2 ions and>3.75 for +3 ions or more (from Sequest HT) were selected.

### Treatment of Cal51 TNBC cells with the exosome biogenesis inhibitor GW4869

GW4869 is a cell permeable, potent inhibitor of the biogenesis of exosomes via the inhibition of *N*-SMase.^56^ Cal51 cells grown in both control and senescence inducing conditions were subsequently treated with control vehicle or GW4869, 5 μM (Sigma-Aldrich, #D1692) for 48 h. Cells were trypsinised and seeded for the SA-β-gal enumeration. Media was collected and EVs isolated with ExoSpin.

### Cell proliferation assay

Cells were seeded at a density of 5 × 10^5^ cells/ml in six-well plates. Cells were treated with PTX for 7 days to induce senescence followed by GW4869 treatment, 5 μM (Sigma-Aldrich, #D1692) for 48 h. Cells were subsequently trypsinised, stained with trypan blue and counted using a haemocytometer at 0, 1, 2, 4, 7, 9, 11 and 14 day timepoints.

### Pathway analysis

Pathway, interaction network and functional enrichment analyses were performed with the proteins identified in EVs using IPA (www.ingenuity.com) software (Ingenuity Systems, Qiagen) and InnateDB (http://www.innatedb.com). *P*-values reported for IPA results are calculated by IPA using a right-sided Fisher exact test for over-representation analysis and Benjamini–Hochberg correction for multiple hypothesis testing correction.

### Statistical analyses

All values are expressed as the mean of three independent experiments±s.e.m. The limit of ±1 skewness was set as normal distribution. Skewed data were transformed to Y=Log(Y) to fit a normal distribution. Results were analysed using the Student’s *t*-test, unless as otherwise stated in the quantitative proteomic profiling by label-free liquid chromatography–MS/MS analysis and pathway analysis sections. Significant differences are defined as *P⩽0.05, **P⩽0.01, ***P⩽0.001.

## Figures and Tables

**Table 1:** List of key proteins identified from MS analysis with an increased abundance in TIS EVs vs control EVs

	*Fold change*	*Anova (P)*
*Annexin*		
Annexin A7	191	0.030
Annexin A3	156	0.011
Annexin A5	15	0.002
Annexin A11	13	0.002
Annexin A2	9	0.001
Annexin A4	9	0.005
Annexin A1	6	0.0001
Annexin A6	6	0.002
		
*ATP dependent*		
Sodium/potassium-transporting ATPase subunit beta-1	633	0.017
CTP synthase 1	209	0.022
26S protease regulatory subunit 6B	27	0.044
Potassium-transporting ATPase alpha chain 2	16	0.004
Sodium/potassium-transporting ATPase subunit alpha-1	11	0.005
Pre-mRNA-splicing factor ATP-dependent RNA helicase	3	0.007
26S protease regulatory subunit 10B	3	0.035
ATP-dependent RNA helicase A	3	0.050
		
*Integrins*		
Integrin alpha-V	n/a (protein was not detected by MS in the control samples)	6.76E-07
Integrin beta-3	6	0.020
Integrin beta-1	5	0.022
		
*Tubulin*		
Tubulin alpha-1C chain	8	0.003
Tubulin beta chain	4	0.040
Tubulin alpha-1A chain	4	0.003
Tubulin beta-4A chain	4	0.007
Tubulin beta-2A chain	4	0.006
Tubulin beta-4B chain	3	0.004
Tubulin beta-3 chain	3	0.002
Tubulin alpha-1B chain	3	0.028
Tubulin beta chain	3	0.022
		
*Rab proteins*		
Ras-related protein Rab-1A	10	0.018
Ras-related protein Rab-5C	5	0.010
Ras-related protein Rab-7a	4	0.021
Ras-related protein Rab-5A	3	0.029
Ras-related protein Rab-11A	3	0.010
Ras-related protein Rab-10	3	0.012
		
*Insoluble SASP factors*		
Collagen alpha-1 (V)	15	0.025
Collagen alpha-2 (IV)	7	0.003
Laminin subunit alpha-5	4	0.025
Laminin subunit beta-1	3	0.018
Fibronectin	2	0.001

Abbreviations: EV, extracellular vesicle; MS, mass spectrometry; SASP, senescence-associated secretory phenotype; TIS, therapeutic induced senescence.
