# Supplementary material for: Protein and chemotherapy profiling of extracellular vesicles harvested from therapeutic induced senescent triple negative breast cancer cells
Source: Oncogenesis. 2017 Oct 9;6(10):e388–. doi: 10.1038/oncsis.2017.82 (PMC5668881; doi:10.1038/oncsis.2017.82)
Supplement: Supplementary Figure 1 [file oncsis201782x1.docx]

**Supplemental Figure 1: Molecular structure of paclitaxel (PTX) (Taxol®)** **and Flutax-2 Oregon Green 488™ paclitaxel. A)** Structure of paclitaxel (Taxol®) [www.paclitaxel.co.uk](http://www.paclitaxel.co.uk)  **B)** Flutax-2 consists of a fluorescent conjugate added to the 7’ Carbon of paclitaxel, which enhances its binding activity to tubulin microfilaments. This paclitaxel analogue is routinely used at 10 times the concentration of paclitaxel as per the manufacturers recommendations <https://www.thermofisher.com/order/catalog/product/P22310>*.*

**Supplemental Figure 2:** Complete ‘*Breast Cancer Regulation by Stathmin 1*’ signalling pathway generated using Ingenuity Pathway Analysis (IPA).
