# Supplementary figures and images for: Protein and chemotherapy profiling of extracellular vesicles harvested from therapeutic induced senescent triple negative breast cancer cells
Source: Oncogenesis. 2017 Oct 9;6(10):e388–. doi: 10.1038/oncsis.2017.82 (PMC5668881; doi:10.1038/oncsis.2017.82)

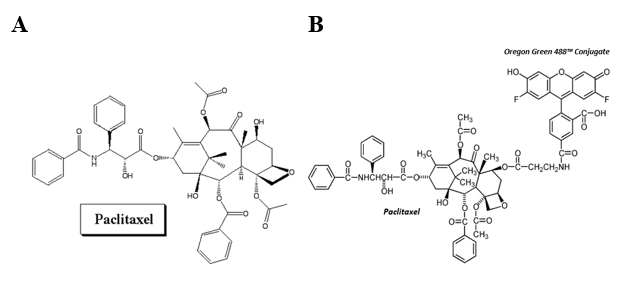

Supplement: Supplementary Figure 2 [file oncsis201782x2.docx]

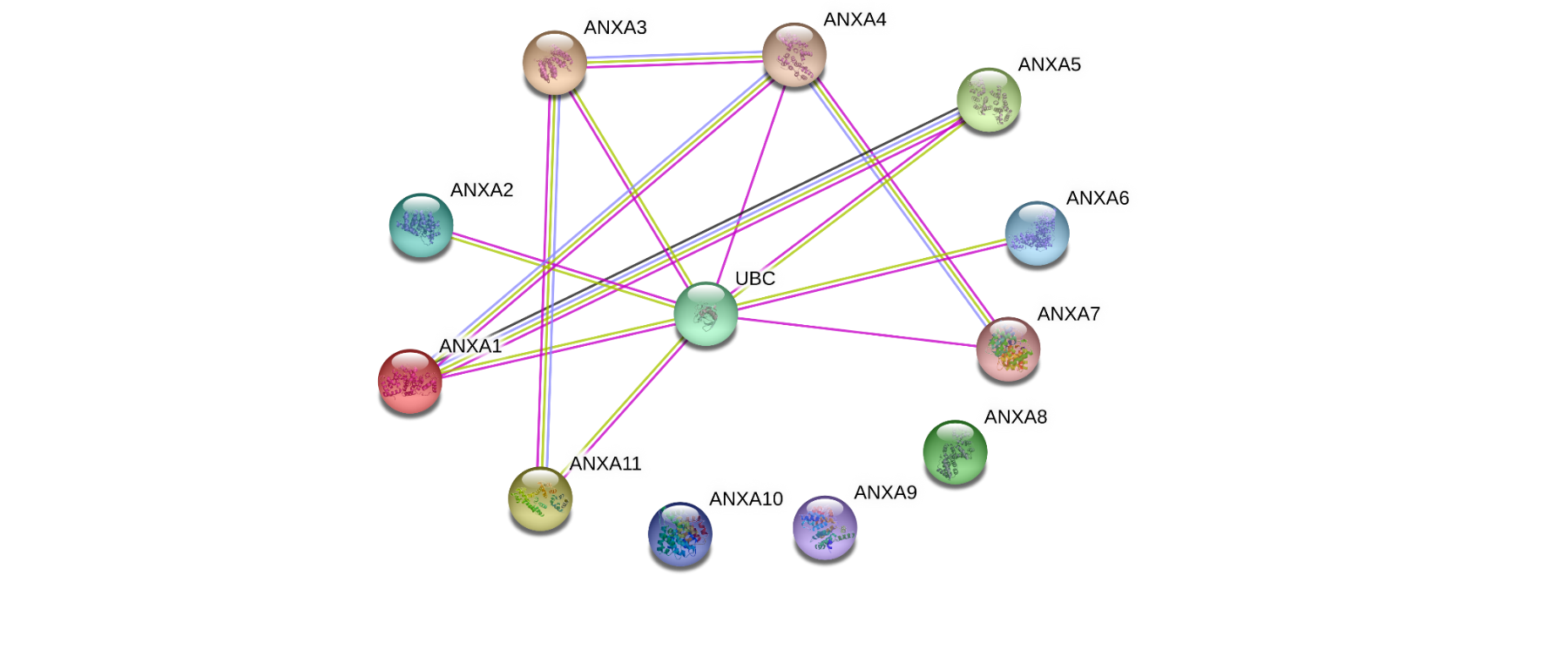
**
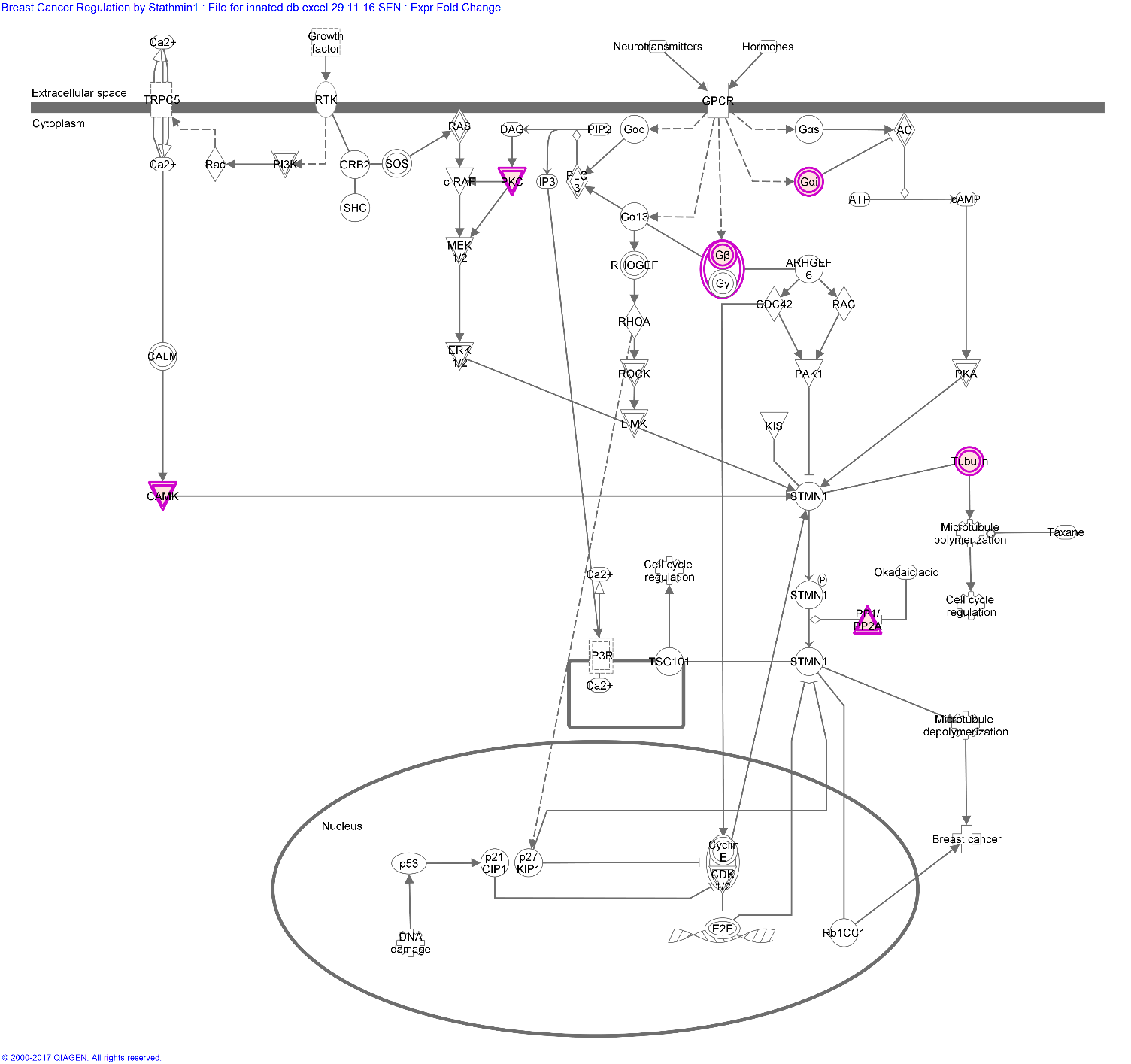
**

Supplement: Supplementary Figure 3 [file oncsis201782x5.docx]
