# Supplementary material for: Protein and chemotherapy profiling of extracellular vesicles harvested from therapeutic induced senescent triple negative breast cancer cells
Source: Oncogenesis. 2017 Oct 9;6(10):e388–. doi: 10.1038/oncsis.2017.82 (PMC5668881; doi:10.1038/oncsis.2017.82)
Supplement: Supplementary Table 1 [file oncsis201782x3.docx]

| Proteins higher in TIS EVs vs control EVs | Accession | Fold change | Anova (p) |
| --- | --- | --- | --- |
| Sodium/potassium-transporting ATPase subunit beta-1 | P05026 | 633 | 0.017 |
| CTP synthase 1 | P17812 | 208 | 0.022 |
| Annexin A7 | P20073 | 191 | 0.030 |
| Annexin A3 | P12429 | 156 | 0.011 |
| Ribosome production factor 2 homologue | Q9H7B2 | 72 | 0.036 |
| Nucleolin | P19338 | 56 | 0.012 |
| Protein kinase C alpha type | P17252 | 50 | 0.043 |
| Myb-binding protein 1A | Q9BQG0 | 49 | 0.007 |
| Semaphorin-3C | Q99985 | 32 | 0.001 |
| DNA-dependent protein kinase catalytic subunit | P78527 | 29 | 0.039 |
| 26S protease regulatory subunit 6B | P43686 | 27 | 0.044 |
| Unconventional myosin-Ic | O00159 | 27 | 0.017 |
| Growth/differentiation factor 15 | Q99988 | 19 | 0.013 |
| Protein S100-A10 | P60903 | 17 | 0.006 |
| Potassium-transporting ATPase alpha chain 2 | P54707 | 16 | 0.004 |
| Collagen alpha-1(IV) chain | P02462 | 15 | 0.025 |
| Annexin A5 | P08758 | 15 | 0.002 |
| Annexin A11 | P50995 | 13 | 0.002 |
| Sodium/potassium-transporting ATPase subunit alpha-1 | P05023 | 11 | 0.005 |
| Calcium/calmodulin-dependent protein kinase type II subunit delta | Q13557 | 11 | 0.026 |
| 4F2 cell-surface antigen heavy chain | P08195 | 11 | 0.006 |
| Glycogen [starch] synthase, muscle | P13807;P54840 | 10 | 0.002 |
| Ras-related protein Rab-1A | P62820 | 10 | 0.018 |
| Nucleolar GTP-binding protein 1 | Q9BZE4 | 9 | 0.046 |
| Annexin A2 | P07355 | 9 | 0.001 |
| Annexin A4 | P09525 | 9 | 0.005 |
| Folate receptor alpha | P15328 | 8 | 0.008 |
| Tubulin alpha-1C chain | Q9BQE3 | 8 | 0.003 |
| L-lactate dehydrogenase A chain | P00338 | 8 | 0.003 |
| Solute carrier family 2, facilitated glucose transporter member 1 | P11166 | 7 | 0.032 |
| Collagen alpha-2(IV) chain | P08572 | 7 | 0.003 |
| Lactadherin | Q08431 | 7 | 0.014 |
| Tissue factor pathway inhibitor 2 | P48307 | 7 | 0.016 |
| 78 kDa glucose-regulated protein | P11021 | 7 | 0.008 |
| Adenine phosphoribosyltransferase | P07741 | 7 | 0.013 |
| Casein kinase II subunit alpha | P68400 | 7 | 0.002 |
| Argininosuccinate synthase | P00966 | 6 | 0.021 |
| Annexin A1 | P04083 | 6 | 0.0001 |
| Coagulation factor X | P00742 | 6 | 0.014 |
| Annexin A6 | P08133 | 6 | 0.002 |
| Integrin beta-3 | P05106 | 6 | 0.020 |
| Major vault protein | Q14764 | 6 | 0.015 |
| Matrilin-2 | O00339 | 6 | 0.0005 |
| Casein kinase II subunit beta | P67870 | 6 | 0.000 |
| 60S ribosomal protein L18 | Q07020 | 6 | 0.006 |
| 60S ribosomal protein L7 | P18124 | 6 | 0.043 |
| Peroxiredoxin-1 | Q06830 | 6 | 0.015 |
| Vinculin | P18206 | 5 | 0.0001 |
| Ras-related protein Rab-5C | P51148 | 5 | 0.010 |
| 116 kDa U5 small nuclear ribonucleoprotein component | Q15029 | 5 | 0.012 |
| Heterogeneous nuclear ribonucleoprotein U | Q00839 | 5 | 0.044 |
| Integrin beta-1 | P05556 | 5 | 0.022 |
| 60S ribosomal protein L18a | Q02543 | 5 | 0.015 |
| Pre-mRNA-processing-splicing factor 8 | Q6P2Q9 | 5 | 0.036 |
| Ezrin | P15311 | 5 | 0.039 |
| Guanine nucleotide-binding protein subunit alpha-12 | Q03113;P63096 | 5 | 0.014 |
| Agrin | O00468 | 5 | 0.015 |
| Ribosomal L1 domain-containing protein 1 | O76021 | 5 | 0.014 |
| Heterochromatin protein 1-binding protein 3 | Q5SSJ5 | 5 | 0.026 |
| Tubulin beta chain | P07437 | 4 | 0.040 |
| 60S ribosomal protein L10a | P62906 | 4 | 0.026 |
| Nucleosome assembly protein 1-like 1 | P55209 | 4 | 0.019 |
| Guanine nucleotide-binding protein G(I)/G(S)/G(T) subunit beta-2 | P62879 | 4 | 0.033 |
| 60S ribosomal protein L13 | P26373 | 4 | 0.030 |
| Tubulin alpha-1A chain | Q71U36 | 4 | 0.003 |
| Tubulin beta-4A chain | P04350;Q3ZCM7 | 4 | 0.007 |
| Tubulin beta-2A chain | Q13885 | 4 | 0.006 |
| T-complex protein 1 subunit beta | P78371 | 4 | 0.016 |
| Histone H2A type 1-B/E | P04908 | 4 | 0.019 |
| Ras-related protein Rap-1A | P62834 | 4 | 0.024 |
| Eukaryotic initiation factor 4A-I | P60842 | 4 | 0.003 |
| Casein kinase II subunit alpha' | P19784 | 4 | 0.007 |
| Transaldolase | P37837 | 4 | 0.030 |
| Fatty acid synthase | P49327 | 4 | 0.035 |
| Keratin, type II cytoskeletal 8 | P05787 | 4 | 0.009 |
| L-lactate dehydrogenase B chain | P07195 | 4 | 0.006 |
| Ras-related protein Rab-7a | P51149 | 4 | 0.021 |
| Laminin subunit alpha-5 | O15230 | 4 | 0.025 |
| Serine/arginine-rich splicing factor 11 | Q05519 | 3 | 0.018 |
| Guanine nucleotide-binding protein G(i) subunit alpha-2 | P04899 | 3 | 0.031 |
| Tubulin beta-4B chain | P68371 | 3 | 0.004 |
| Tubulin beta-3 chain | Q13509 | 3 | 0.002 |
| Isoleucine--tRNA ligase, cytoplasmic | P41252 | 3 | 0.021 |
| 26S protease regulatory subunit 10B | P62333 | 3 | 0.035 |
| 60S ribosomal protein L3 | P39023 | 3 | 0.018 |
| Transgelin-2 | P37802 | 3 | 0.008 |
| Ras-related protein Rab-5A | P20339 | 3 | 0.029 |
| Acyl-protein thioesterase 1 | O75608 | 3 | 0.023 |
| 60S ribosomal protein L3-like | Q92901 | 3 | 0.034 |
| Glutathione S-transferase P | P09211 | 3 | 0.013 |
| Putative nucleoside diphosphate kinase | O60361 | 3 | 0.001 |
| Heat shock 70 kDa protein 4 | P34932 | 3 | 0.007 |
| Serine/arginine-rich splicing factor 2 | Q01130 | 3 | 0.015 |
| Serine/arginine-rich splicing factor 7 | Q16629 | 3 | 0.031 |
| 14-3-3 protein zeta/delta | P63104 | 3 | 0.007 |
| Ras-related protein Rab-11A | P62491 | 3 | 0.010 |
| Basigin | P35613 | 3 | 0.032 |
| Pre-mRNA-splicing factor ATP-dependent RNA helicase DHX15 | O43143 | 3 | 0.007 |
| Fructose-bisphosphate aldolase A | P04075 | 3 | 0.036 |
| Thrombospondin-1 | P07996 | 3 | 0.001 |
| Core histone macro-H2A.1 | O75367 | 3 | 0.028 |
| Ras-related protein Rab-10 | P61026;P20340;P59190;Q15286;Q9H082 | 3 | 0.012 |
| Ribose-phosphate pyrophosphokinase 1 | P60891 | 3 | 0.032 |
| 60S ribosomal protein L23 | P62829 | 3 | 0.033 |
| Tubulin alpha-1B chain | P68363 | 3 | 0.028 |
| Histone H2A type 1-C | Q93077 | 3 | 0.021 |
| Triosephosphate isomerase | P60174 | 3 | 0.008 |
| Guanine nucleotide-binding protein G(I)/G(S)/G(T) subunit beta-1 | P62873 | 3 | 0.046 |
| Nucleolar RNA helicase 2 | Q9NR30 | 3 | 0.030 |
| Coatomer subunit beta'] | P35606 | 3 | 0.040 |
| Tubulin beta-1 chain | Q9H4B7 | 3 | 0.022 |
| 60S ribosomal protein L22 | P35268 | 3 | 0.027 |
| Serine/threonine-protein phosphatase PP1-alpha catalytic subunit | P62136 | 3 | 0.045 |
| ATP-dependent RNA helicase A | Q08211 | 3 | 0.050 |
| Laminin subunit beta-1 | P07942 | 2 | 0.018 |
| Keratin, type I cytoskeletal 18 | P05783 | 2 | 0.015 |
| Transketolase | P29401 | 2 | 0.008 |
| Glyceraldehyde-3-phosphate dehydrogenase | P04406 | 2 | 0.001 |
| Clathrin heavy chain 1 | Q00610 | 2 | 0.008 |
| Ras-related protein Ral-A | P11233 | 2 | 0.048 |
| 40S ribosomal protein S6 | P62753 | 2 | 0.029 |
| Eukaryotic initiation factor 4A-III | P38919 | 2 | 0.028 |
| Phosphoglycerate mutase 1 | P18669 | 2 | 0.049 |
| CD81 antigen | P60033 | 2 | 0.044 |
| Alpha-enolase | P06733;P13929 | 2 | 0.004 |
| 40S ribosomal protein S8 | P62241 | 2 | 0.023 |
| Actin, cytoplasmic 1 | P60709 | 2 | 0.004 |
| Adenylyl cyclase-associated protein 1 | Q01518 | 2 | 0.023 |
| 60S ribosomal protein L5 | P46777 | 2 | 0.040 |
| Actin-related protein 2 | P61160 | 2 | 0.040 |
| 60S ribosomal protein L19 | P84098 | 2 | 0.022 |
| 40S ribosomal protein S16 | P62249 | 2 | 0.046 |
| Vitronectin | P04004 | 2 | 0.042 |
| 40S ribosomal protein S3 | P23396 | 2 | 0.042 |
| Signal peptide, CUB and EGF-like domain-containing protein 3 | Q8IX30 | 2 | 0.018 |
| Histone H2B type 1-H | Q93079 | 2 | 0.011 |
| Transcription intermediary factor 1-beta | Q13263 | 2 | 0.044 |
| Beta-2-glycoprotein 1 | P02749 | 2 | 0.027 |
| Phosphoglycerate kinase 1 | P00558 | 2 | 0.034 |
| GTP-binding nuclear protein Ran | P62826 | 1 | 0.022 |
| Fibronectin | P02751 | 1 | 0.001 |
| Basement membrane-specific heparan sulfate proteoglycan core protein | P98160 | 1 | 0.359 |
| Histone H4 | P62805 | 1 | 0.511 |
