# Supplementary material for: Protein and chemotherapy profiling of extracellular vesicles harvested from therapeutic induced senescent triple negative breast cancer cells
Source: Oncogenesis. 2017 Oct 9;6(10):e388–. doi: 10.1038/oncsis.2017.82 (PMC5668881; doi:10.1038/oncsis.2017.82)
Supplement: Supplementary Table 1 [file oncsis201782x4.docx]

| Proteins higher in abundance in control EVs vs TIS-derived EVs | Accession | Fold change | Anova (p) |
| --- | --- | --- | --- |
| D-3-phosphoglycerate dehydrogenase | O43175 | 169 | 0.042 |
| Signal transducing adapter molecule 1 | Q92783 | 56 | 0.016 |
| Left-right determination factor 1 | O75610 | 52 | 0.016 |
| Hepatocyte growth factor-regulated tyrosine kinase substrate | O14964 | 12 | 0.018 |
| Protein TFG | Q92734 | 10 | 0.048 |
| Histone H1.2 | P16403 | 7 | 0.040 |
| Serine/arginine repetitive matrix protein 1 | Q8IYB3 | 6 | 0.013 |
| T-complex protein 1 subunit epsilon | P48643 | 5 | 0.039 |
| Collagen alpha-2(I) chain | P08123 | 4 | 0.004 |
| 40S ribosomal protein S30 | P62861 | 4 | 0.002 |
| Histone H2A type 2-A | Q6FI13 | 4 | 0.026 |
| Regulator of chromosome condensation | P18754 | 4 | 0.026 |
| Keratin, type I cytoskeletal 10 | P13645 | 4 | 0.028 |
| Nuclease-sensitive element-binding protein 1 | P67809 | 3 | 0.037 |
| Histone H3.1t | Q16695 | 3 | 0.002 |
| Clusterin | P10909 | 3 | 0.019 |
| Galectin-3-binding protein | Q08380 | 3 | 0.017 |
| Putative small nuclear ribonucleoprotein G-like protein 15 | A8MWD9 | 3 | 0.039 |
| Midkine | P21741 | 3 | 0.005 |
| 40S ribosomal protein S10 | P46783 | 2 | 0.024 |
| Desmocollin-2 | Q02487 | 2 | 0.003 |
| Small nuclear ribonucleoprotein-associated proteins B and B' | P14678 | 2 | 0.021 |
| T-complex protein 1 subunit alpha | P17987 | 2 | 0.033 |
| 60S ribosomal protein L29 | P47914 | 2 | 0.045 |
| Complement component C9 | P02748 | 2 | 0.001 |
| Transitional endoplasmic reticulum ATPase | P55072;Q8IYT4 | 2 | 0.020 |
| Non-histone chromosomal protein HMG-17 | P05204 | 2 | 0.007 |
| T-complex protein 1 subunit gamma | P49368 | 2 | 0.034 |
| Small nuclear ribonucleoprotein Sm D2 | P62316 | 2 | 0.028 |
| Non-histone chromosomal protein HMG-14 | P05114 | 2 | 0.015 |
| Fibulin-1 | P23142 | 1 | 0.022 |
| Apolipoprotein M | O95445 | 1 | 0.024 |
